# Praziquantel degradation in marine aquarium water

**DOI:** 10.7717/peerj.1857

**Published:** 2016-04-04

**Authors:** Amber Thomas, Matthew R. Dawson, Helen Ellis, M. Andrew Stamper

**Affiliations:** 1 The Seas, Epcot, Walt Disney World Resort, Disney’s Animals, Science and Environment, Lake Buena Vista, FL, United States; 2 Georgia Aquarium, Atlanta, GA, United States

**Keywords:** Praziquantel, Aquarium, Treatment, Drug, Water quality, Therapy, Microbial

## Abstract

Praziquantel (PZQ) is a drug commonly utilized to treat both human schistosomiasis and some parasitic infections and infestations in animals. In the aquarium industry, PZQ can be administered in a “bath” to treat the presence of ectoparasites on both the gills and skin of fish and elasmobranchs. In order to fully treat an infestation, the bath treatment has to maintain therapeutic levels of PZQ over a period of days or weeks. It has long been assumed that, once administered, PZQ is stable in a marine environment throughout the treatment interval and must be mechanically removed, but no controlled experiments have been conducted to validate that claim. This study aimed to determine if PZQ would break down naturally within a marine aquarium below its 2 ppm therapeutic level during a typical 30-day treatment: and if so, does the presence of fish or the elimination of all living biological material impact the degradation of PZQ? Three 650 L marine aquarium systems, each containing 12 fish (French grunts: *Haemulon flavolineatum*), and three 650 L marine aquariums each containing no fish were treated with PZQ (2 ppm) and concentrations were measured daily for 30 days. After one round of treatment, the PZQ was no longer detectable in any system after 8 (±1) days. The subsequent two PZQ treatments yielded even faster PZQ breakdown (non-detectable after 2 days and 2 ± 1 day, respectively) with slight variations between systems. Linear mixed effects models of the data indicate that day and trial most impact PZQ degradation, while the presence of fish was not a factor in the best-fit models. In a completely sterilized marine system (0.5 L) PZQ concentration remained unchanged over 15 days, suggesting that PZQ may be stable in a marine system during this time period. The degradation observed in non-sterile marine systems in this study may be microbial in nature. This work should be taken into consideration when providing PZQ bath treatments to marine animals to ensure maximum drug administration.

## Introduction

Though the exact mechanism of its functionality is largely unknown (Cioli et al., 2014; Olliaro, Delgado-Romero & Keiser, 2014), praziquantel (PZQ) has been utilized in the treatment of human schistosomiasis since 1984 (Olliaro, Delgado-Romero & Keiser, 2014) and due to its efficacy, cost, safety and convenience of dispersal it has remained the primary treatment for this condition worldwide (Cioli et al., 2014). It is well documented that PZQ is also useful in the treatment of other parasitic infections (Chai, 2013) including those that parasitize animals (Chisholm & Whittington, 2002; Forwood, Harris & Deveney, 2013; Iles, Archdeacon & Bonar, 2012; Ishimaru et al., 2013; Janse & Borgsteede, 2003; Masimirembwa, Naik & Hasler, 1993; Mitchell & Darwish, 2009; Mitchell, 2004; Schmahl & Mehlhorn, 1985; Schmahl & Taraschewski, 1987; Shirakashi et al., 2012; Steiner et al., 1976; Tubbs, Mathieson & Tingle, 2008; Tubbs & Tingle, 2006a; Ward, 2007; Yamamoto et al., 2011).

In the aquarium industry, PZQ has been used to treat trematode, cestode and monogene infestations in both freshwater (Forwood, Harris & Deveney, 2013; Iles, Archdeacon & Bonar, 2012; Mitchell & Darwish, 2009; Mitchell, 2004; Santamarina et al., 1991) and marine fish and elasmobranchs (Chisholm & Whittington, 2002; Ishimaru et al., 2013; Janse & Borgsteede, 2003; Shirakashi et al., 2012; Thoney, 1990; Tubbs & Tingle, 2006a; Tubbs & Tingle, 2006b; Yamamoto et al., 2011). Though this drug can be administered to fish orally (Forwood, Harris & Deveney, 2013; Janse & Borgsteede, 2003; Tubbs, Mathieson & Tingle, 2008; Tubbs & Tingle, 2006a; Tubbs & Tingle, 2006b), it is also common for it to be dissolved in the water in which the infested animal lives (Chisholm & Whittington, 2002; Forwood, Harris & Deveney, 2013; Iles, Archdeacon & Bonar, 2012; Mitchell & Darwish, 2009; Mitchell, 2004). “Bath” treatments can range in duration from several minutes to several days to effectively remove parasites. The common treatments generally involve either short-term baths at a high PZQ concentration (10–100 ppm PZQ for minutes–hours Noga, 2011) or long-term baths at low PZQ concentrations (1–2 ppm for days–weeks). In order to ensure the maximum parasite removal has occurred with the least amount of stress on the animal, optimal doses of PZQ must be maintained throughout both short and long-term treatments.

It has long been assumed within the aquarium industry that PZQ is stable in a marine aquarium throughout this treatment period, and PZQ would therefore have to be removed by a chemical filtration unit (i.e., a carbon filter), chemical breakdown through ozone/UV disinfection or a complete water change after the bath treatment had finished. However, the same assumption existed for formalin, another drug used in marine aquarium systems, but a recent study indicated that these assumptions for formalin were largely false. Formalin is often prescribed as a 5-day bath treatment, but investigation showed that concentrations decreased below detectable limits in as few as 4 h (Knight, Boles & Stamper, in press). While some observations of a similar breakdown of PZQ exist (Crowder & Charanda, 2004; Marrero & Ellis, 2014), to our knowledge, no controlled study has been conducted. This study aimed to answer three questions: (1) How long does PZQ last in a closed, marine aquarium system? (2) Does the presence of fish affect PZQ degradation? (3) Does the elimination of living biological material impact PZQ degradation?

## Methods

This study utilized six, recirculating 650 L systems. The water in each system was mechanically filtered through a 50 µm pre-filter and a 20 µm pleated cartridge filter. It was biologically filtered through a trickle filter/sump containing 29.5 L of 1-inch bio barrels and bio-balls (Pentair Aquatic Eco-Systems, Apopka, FL, USA) and then passed through two 250 L aquarium tanks. The biological filter in all systems was started with the same bottle of Fritz-Zyme 9, Live Nitrifying Bacteria (Mesquite, TX, USA). All systems were filled with 30–32 ppt artificial salt water, which was made using Instant Ocean^®^ (Spectrum Brands, Blacksburg, VA, USA) and carbon-filtered potable water.

French grunts (*Haemulon flavolineatum)* were placed in three, randomly selected systems, such that there were six fish in each tank and twelve fish in each system. All fish were captive-bred at the University of Florida’s Tropical Aquaculture Lab and were approximately 22 g when introduced to the study. All fish were fed at approximately 6% of their body weight in aquatic gel (5AB0 MTLS Aquatic Gel; Mazuri, St. Louis, MO, USA) daily. The remaining three systems were maintained as “No Fish Systems” systems and were “fed” 1.0 ppm of (NH_4_)_2_SO4 (VWR, ACS Grade, Radnor PA, USA) daily, based on a calculated approximation of ammonia production in the fish systems.

Each system was dosed with PZQ (P4668; Sigma Aldrich, St. Louis, MO, USA) at 2 ppm concentration, by mechanical dissolution of chemical inside a 350 µm net, added to the system sump near the pump intake. At a flow rate of 34 L minute^−1^, the water in these systems turned over completely every 20 min. After 90 min, the whole system had been turned over 4.5 times, allowing the chemical to be evenly distributed through each system. A 500 mL water sample was collected with polypropylene bottles from each system ninety minutes after the initial PZQ addition and is referred to as the “Day 0” sample throughout this study. Every subsequent day after initial PZQ addition, another 500 mL water sample was collected from each system for a total of 30 days. Immediately after collection, these samples were stored in a cryofreezer (−70 °C) until further analysis could be completed. During the study period, no UV or carbon filters were used, and no water changes were conducted. Daily water chemistries (temperature, salinity, pH, dissolved oxygen) and weekly chemistries (total ammonia as nitrogen, nitrite levels and alkalinity) were taken to ensure the safety of the animals involved in the study. Temperature was maintained at 28 ± 0.7 °C. Salinity was 32 ± 2 ppt for all systems across all treatments. Dissolved oxygen was maintained at 6.9 ± 0.5 mg L^−1^ O_2_ and pH remained at 8.01 ± 0.25.

After the 30-day study period, the system water was run through the carbon filter for four days to remove any potential PZQ still present. The carbon filters were then removed, and the systems were maintained normally for 14 days as a washout period. A 20% water change was then performed to prepare for the next trial of the experiment. Trials 2 and 3 were set up exactly the same way as previously mentioned (Table 1).

**Table 1:** Experimental Setup. Overview of the setup for all 6 trials involved in this study.

Trial	Setup	Duration of experiment	Experimental Factors Involved
1	Six 650 L systems	30 days	3 systems had 12 Fish (i.e., “Fish Systems”)
			3 systems had 0 Fish (i.e., “No Fish Systems”)
2	Six 650 L systems	30 days	3 systems had 12 Fish (i.e., “Fish Systems”)
			3 systems had 0 Fish (i.e., “No Fish Systems”)
3	Six 650 L systems	30 days	3 systems had 12 Fish (i.e., “Fish Systems”)
			3 systems had 0 Fish (i.e., “No Fish Systems”)
4	Six 650 L systems	30 days	No Fish in any system. All systems dosed with 75 ppm Cl^−^ (bleach) 48 h before start of dosing
5	Six 650 L systems	30 days	No Fish in any system. All systems dosed with 200 ppm Cl^−^ (bleach) 48 h before start of dosing
6 (Sterile)	Three 500 mL plastic containers	15 days	All equipment completely sterilized before start of experiment (Called “Sterile” Trial)

Trial 4 of this experiment was used as the control, and thus required that no fish or microorganisms be present in the systems. All fish were removed from the systems, and following 3 weeks of carbon filtration, all systems were bleached (75 ppm Cl^−^). After 24 h, the appropriate amount of sodium thiosulfate (100 g/system) was added to each system to neutralize the bleach. After another full 24 h, PZQ was added to the system (2 ppm) and the sample collection process continued as it had for the first three trials.

Trial 5 of this experiment was used as a second control in which all six of the systems were bleached at a higher concentration (200 ppm) to ensure complete eradication of any microorganisms. After 24 h the appropriate amount of sodium thiosulfate (220 g) was added to neutralize the bleach and another 24 h later, PZQ (2 ppm) was added to each system and the sample collection process continued as previously described.

To test the degradation of PZQ in a completely sterilized system, this process was repeated in a 500 mL sample bottle. Any lab-ware that would come into contact with this “system” was sterilized for at least 20 min at 120 °C. Seawater from the same supply that was used in the first five trials of this experiment, but had never been exposed to PZQ was also sterilized for 20 min at 120 °C. Exactly 5 mL of 200 ppm PZQ stock solution was added to 495 mL of sterilized seawater in each of six 500-mL sample bottle and covered. Three of the six samples were put into the freezer (−70 °C) 90 min after PZQ addition, while the remaining three samples remained in a chemical hood at room temperature for 15 days and was then placed in the same freezer.

### Ethics statement

This project was approved by Disney’s Animal Care and Welfare Committee (IR #1102). PZQ concentrations never exceeded prolonged immersion therapeutic levels (2 ppm) for the fish in the study and both water quality and fish health (i.e., food consumption, lack of wounds, normal movement and socialization etc.) were monitored on a daily basis.

### Extractions

To extract the PZQ from the water samples, samples were systematically removed from the deep freezer and allowed to thaw at room temperature overnight. The following morning, samples were extracted using a C-8 disk (Sigma Aldrich) and vacuum filtration as described by the C-8 disk instructions. Methanol (ACS spectrophotometric grade; Sigma Aldrich) and nanopure water were used to condition the C-8 disk before the sample was poured through. Once the sample was filtered, the collection vial was added to the vacuum apparatus, and 10 mL of acetonitrile (40%; Sigma Aldrich) was vacuumed through the filter three times into the collection vial for a total of 30 mL of acetonitrile solution (Crowder & Charanda, 2004). This eluent was retained and stored in a refrigerator (4.4 °C) until it was shipped with ice packs to the Georgia Aquarium for high performance, liquid chromatography (HPLC) analysis. Due to monetary constraints, it was impossible to analyze every sample collected (a total of 180 samples per treatment). Instead, a sample from every 3rd day from each system was analyzed initially. Once the time scale in which concentrations decreased below detectable limits was known, few additional samples were analyzed for finer resolution.

### HPLC analysis

Aliquots of extracted samples were placed in 2 mL extraction vials and analyzed on a Dionex Ultimate 3000 UHPLC. An eluent solution of 40% acetonitrile was pumped at a 1.6 ml/min flow rate onto a Whatman Partisil 5 ODS – 3 4.6 × 250 mm analytical column warmed to 30 °C. The samples were injected by the auto sampler warmed to 30 °C and analyzed by the UV dector at 210 nm. Chromatograms were produced and analyzed by the chromeleon 6.8 software. A five point standard curve ranging from 1 mg/L to 50 mg/L, an extracted known and a known were used as the quality control for each batch.

### Statistics

To account for any errors associated with repeatedly sampling from the same systems and the unbalanced data sets between systems that resulted from our search for finer resolution, linear mixed effects models were used to determine the relationships between the parameters in this study. In all models, ‘system’ was used as a random factor, PZQ concentration was the response variable and treatment (presence of fish or chlorine bleach), day and trial were the explanatory variables. All explanatory variables were modeled independently and with all interactive effects. Model estimates were obtained using the lme4 package (Bates et al., 2014) in R Core Team (2014) using restricted likelihood estimates (REML). The ‘anova’ function in lme4 was used to compare models using the AIC (Akaike’s Information Criterion), such that the model with the smallest AIC was the best fit model for the data. The ‘anova’ function in the lmerTest package (Kuznetsova, Brockhoff & Christensen, 2014) was used to compute degrees of freedom and *p*-values of the factors involved in the best fit models.

## Results

**Figure 1:**
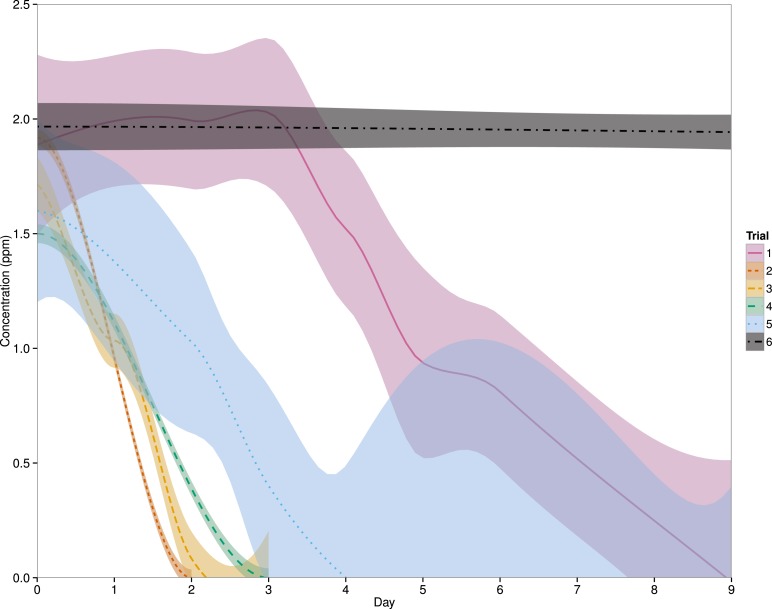
Effects of day and trial on PZQ concentrations. The solid, dashed and dotted lines represent the LOESS smoothing of the data from this study with a shaded area representing the 95% confidence interval. Treatments (fish vs. no fish systems) have been pooled as the best fit model suggested that ‘Treatment’ was not a significant factor. Descriptions of each trial can be found in Table 1. For Trials 1–5, *n* = 6 systems and for Trial 6, *n* = 3.

Eight linear mixed effects models were compared to determine the best fit model for the data (Table 2) and a model which includes ‘system’ as a random effect and an interaction between ‘day’ and ‘trial’ was found to be the best fit (Fig. 1). Since ‘treatment’ was not included in this model, the ‘fish’ and ‘no fish’ system data will be pooled for the remainder of this manuscript. After the first administration of PZQ (Trial 1), the chemical was no longer detected in the system water by an average (±SD) of 8 ± 1 days. PZQ degradation was faster in Trial 2, with concentrations declining below detectable limits by 2 days, and a similar pattern was seen in Trial 3, where PZQ was non-detectable by 2 ± 1 day. Both Trials 4 and 5 were intended to be utilized as controls in this experiment, and thus no PZQ degradation was expected. However, PZQ was no longer at a detectable limit in the systems by 3 days and 5 ± 3 days, respectively. In the completely sterile system, where PZQ was left completely undisturbed in 500 mL plastic containers, the PZQ concentrations remained unchanged over a 15 day time-span (one-way ANOVA, *p* = 0.523) (Fig. 1).

**Table 2:** Summary of linear mixed effects models used to analyze factors that could influence PZQ concentration in this study. Values represent degrees of freedom (Df) of the model and Akaike’s Information Criterion (AIC).

Model	Df	AIC
Random effects	3	284.11
Day	4	274.50
Treatment	6	274.07
Trial	8	270.57
Day × Treatment	10	245.27
**Day** × **Trial**	**14**	139.10^*^
Treatment × Trial	11	276.43
Day × Trial × Treatment	20	148.44

**Notes.**

* Indicates the best fit model based on AIC selection process.

## Discussion

This study had 4 major findings: (1) PZQ is stable for at least 15 days in a completely sterile environment; (2) PZQ, even in a naïve aquarium system, degrades below detectable limits in less than 9 days; (3) A second introduction of PZQ to an aquarium system results in faster PZQ degradation rates than the first treatment, (4) Fish do not impact the degradation of PZQ in a marine aquarium system.

First, in our sterile trial, in which sterilized water in three separate, 500-mL polypropylene sample bottles were dosed from the same PZQ stock solution, the drug concentration remained statistically unchanged for 15 days, suggesting that it is stable under these conditions. Thus, any degradation observed in the other trials of this experiment is not thought to be attributed to natural PZQ instability in seawater, during this time frame.

Second, the presence of fish was not a factor in the best fit models for these data, suggesting that the presence of fish did not significantly impact the reduction of PZQ in the water. However, this is not to say that marine fish are incapable of extracting PZQ from water. Rockfish (*Sebastes schlegeli*) treated with a high concentration, short-term PZQ bath (100 ppm for 4 min) were found to contain PZQ in their plasma and muscles for up to 72 h after treatment. However, these concentrations were minimal (maximum 5.96 µg mL^−1^ plasma and 0.49 µg g^−1^ muscle) (Kim, Kim & Kim, 2001). Similarly, when kingfish (*Seriola lalandi*) were treated with oral PZQ, the plasma concentrations of the drug reached non-detectable limits within 24 h of administration (Tubbs & Tingle, 2006a), further suggesting that the drug does not accumulate in the animal’s body. Though PZQ concentrations in skin, blood or plasma were not measured in this study, the lack of any notable difference in PZQ concentrations in the water between systems with fish and those without fish indicate that the fish did not accumulate or remove a significant amount of the drug from the water, and thus are not likely the cause of its concentration decline. A similar trend was seen in the degradation of formalin in seawater, where doubling the fish densities in marine systems did not affect the rate of formalin degradation and was therefore not thought to be a contributing factor (Knight, Boles & Stamper, in press).

Next, we observed PZQ degradation below detectable limits in every system and every experimental trial in this experiment within nine days of treatment. Each of these systems used the same mechanical filtration units, and thus any impact of mechanical filtration on degradation rate would be expected to be more similar between systems and trials. Instead, the overall degradation rate varied between trials such that the first exposure to PZQ showed the slowest rate of degradation, reaching non-detectable limits by nine days. Every subsequent trial exhibited PZQ degradation to the similar level by four days. Though this increase could be the result of mechanical filtration, it seems unlikely that the degradation rate in each system would remain consistent within a trial but not between trials.

The increase in rate could be due to an increase in microbial populations (bacteria, protists, algae, phytoplankton, cyanobacteria etc.) which may be able to utilize PZQ as an energy source after the first exposure to the drug. This same pattern of rate increase after the first dosing was also seen when a 21.5 million L salt water aquarium was dosed with 2 ppm PZQ (Crowder & Charanda, 2004) suggesting that this is not merely a phenomena of small tank volumes. This variable breakdown rate was also seen repeatedly in the degradation of formalin in seawater (Adroer et al., 1990; Dickerson & Heukelekian, 1950; Kaszycki & Kołoczek, 2000; Pedersen, Pedersen & Sortkjær, 2007) which was theorized as microbial degradation (Knight, Boles & Stamper, in press). To further support our hypothesis that the PZQ breakdown can be attributed to microbial breakdown, PZQ concentrations remained the same in our sterile systems, as previously described, indicating that PZQ concentration can remain consistent in seawater for at least 15 days. Overall, this indicates that PZQ does breakdown in marine ecosystems, yet, there is no evidence to suggest that breakdown observed in this study is natural for this chemical during this time frame, or that it can be attributed to mechanical filtration or removal by marine fish (*H. flavolineatum*), thus indicating the microbiota may be the cause. It is unclear at this time which, if any, microorganisms can metabolize PZQ from seawater, but any further research into this subject would greatly aid the field.

In trials 4 and 5 of this experiment, fish were removed from the systems and each system was bleached at 75 ppm and 200 ppm Cl^−^, respectively. These trials were intended to act as controls by removing any biological material from the systems. However, PZQ still degraded within three days of treatment in these trials, indicating that either the breakdown is mechanical or that the bleach was not successful at removing all living microorganisms from the system. We discuss the unlikelihood of this degradation being an effect of mechanical filtration previously, and thus are left with the conclusion that microorganisms that can metabolize PZQ were not completely removed by the bleaching treatment.

Within a marine aquarium, many microorganisms including bacteria, fungi, and protozoans inhabit a biofilm on surfaces within the aquarium system which is semi-protected from non-ideal environmental conditions with an extracellular polymeric matrix. Some biofilms have been described as so hydrophobic that they repel water and other liquids as well as Teflon (Epstein et al., 2011) making them impenetrable and unsusceptible to bleach. Though, some biofilms have been shown to be susceptible to killing by chlorine bleach at a concentration of 10 ppm after 127 min, or after only 12 min at a concentration of 90 ppm (Grobe, Zahller & Stewart, 2002). If the microorganisms in the biofilm survived the chlorine bleach treatment, they could then engage in cell dispersal after the chlorine had been removed, releasing many cells at once or few cells continuously to aid in re-colonization of the aquarium (for review on biofilm cell dispersion, see Solano, Echeverz & Lasa, 2014). In this study, the biofilm is thought to have survived 24 h at a concentration of 200 ppm Cl^−^ and then released microorganisms back into the systems that were able to degrade PZQ in three days. The dynamics and likelihood of this hypothesis are unknown at this time, but studies investigating the rate at which a biofilm can re-colonize a system with microorganisms that can metabolize PZQ are needed to substantiate this claim.

Ultimately, this study suggests that if marine aquarium systems are being treated with a bath of PZQ, it is important that the drug concentration is monitored throughout the treatment to ensure that therapeutic levels are being maintained. Further, in mature systems and systems that have experienced PZQ in the past, the rate of degradation may only increase, leading to non-detectable levels after as few as two days. Future studies which aid in a better understanding of the minimum effective dose for treating various parasitic infections and infestations would greatly benefit this field and PZQ users worldwide.

## Additional Information and Declarations

### Data Availability

The following information was supplied regarding data availability: Figshare: http://dx.doi.org/10.6084/m9.figshare.1608301
and http://figshare.com/s/0831c3848edb11e5a4cc06ec4b8d1f61.
